# Supplementary figures and images for: Reconstructing the dietary habits and trophic positions of the Longipterygidae (Aves: Enantiornithes) using neontological and comparative morphological methods
Source: PeerJ. 2023 Mar 27;11:e15139. doi: 10.7717/peerj.15139 (PMC10062354; doi:10.7717/peerj.15139)

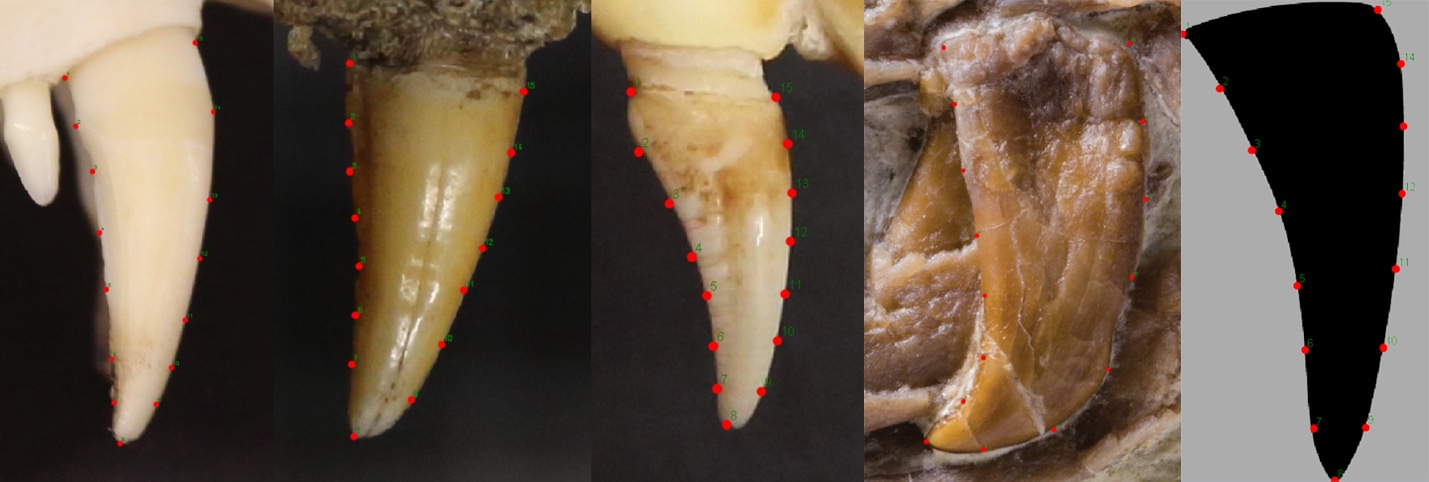

Supplement: Supplemental Information 1 — Three landmarks and two curves measured the morphological changes of the mesial and distal tooth margins in sampled taxa. From left to right, Artictus binturong, Caiman niger, Pteropus conspicillatus, Longipteryx chaoyangensis, “vectorized” Rhinolophus acuminatus. See Supplemental Information for landmarking protocols and taxon used. [file peerj-11-15139-s001.jpg]

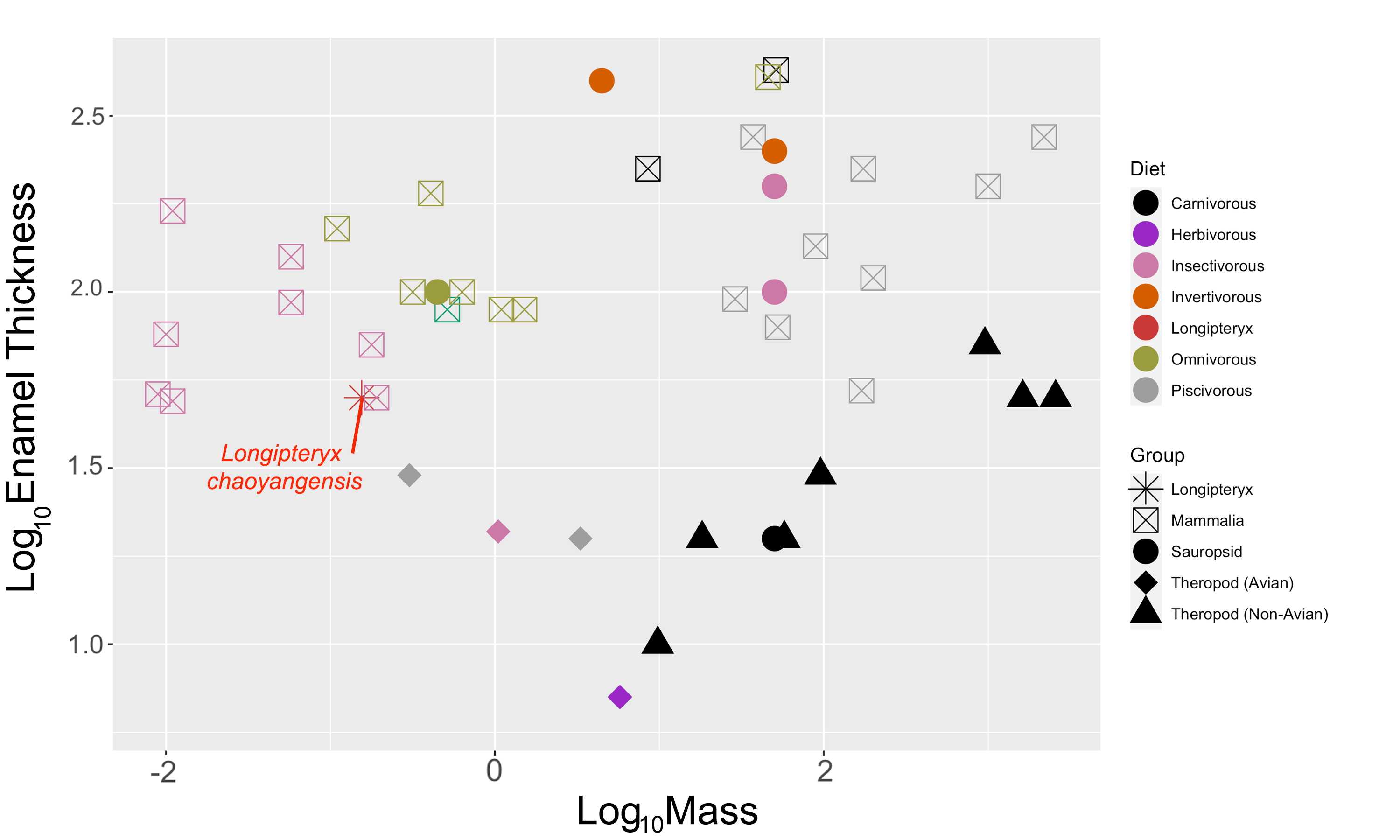

Supplement: Supplemental Information 2 — With limited enamel thickness values available in the current literature, Longipteryx chaoyangensis plots near members of Chiroptera (mammals), and above some non-avian theropods and piscivorous avian theropods. [file peerj-11-15139-s002.png]

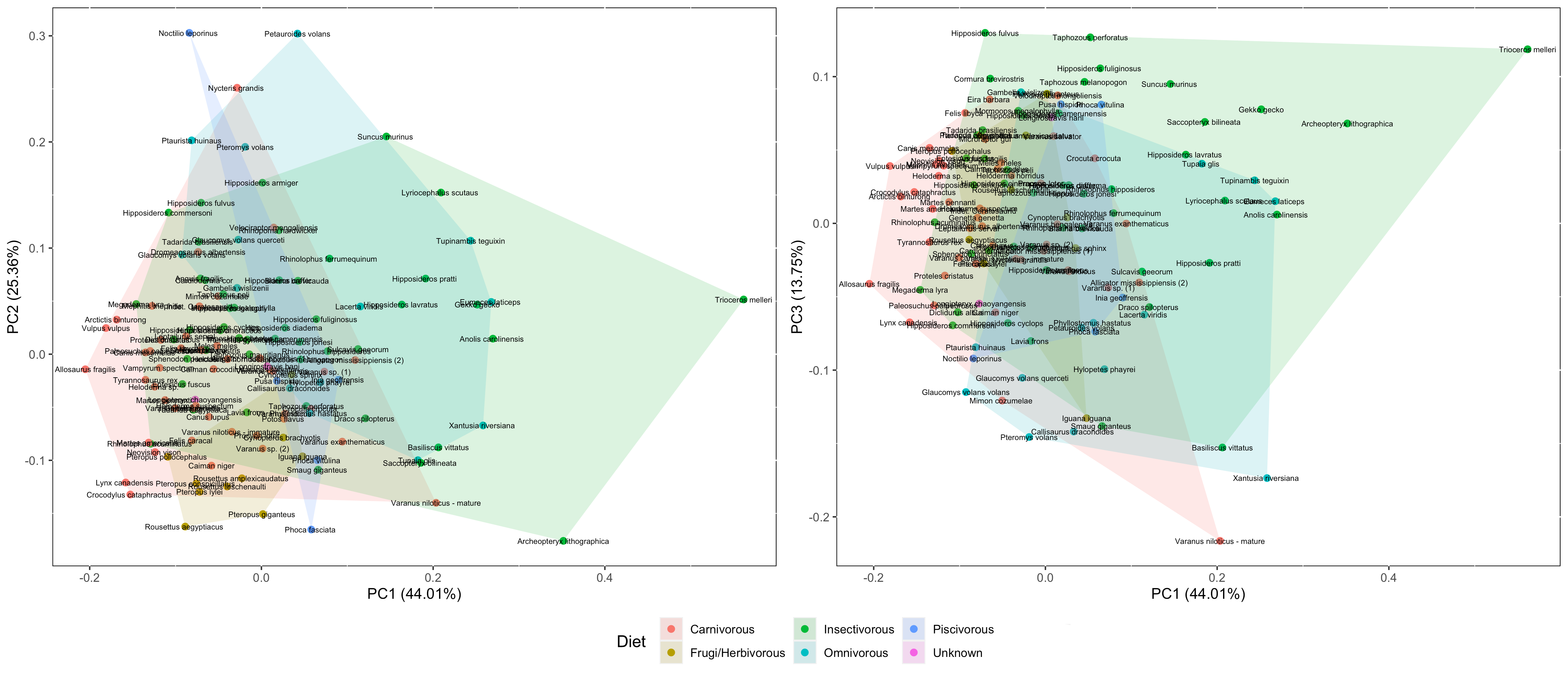

Supplement: Supplemental Information 3 — Using five different dietary preferences and grouping using convex hulls, the morphologically similar teeth are shown compared to Longipteryx chaoyangensis and Longirostravis hani. Interestingly, the tooth morphologies of animalivorous (e.g., insectivorous, piscivorous, carnivorous) taxa show much overlap. Factors such as the size of the predator, the ability or inability to orally process prey, and the strategy of prey acquisition can account for the differences and similarities between taxa. Both longipterygids are labeled in each analysis and represented by an asterisk. [file peerj-11-15139-s003.png]

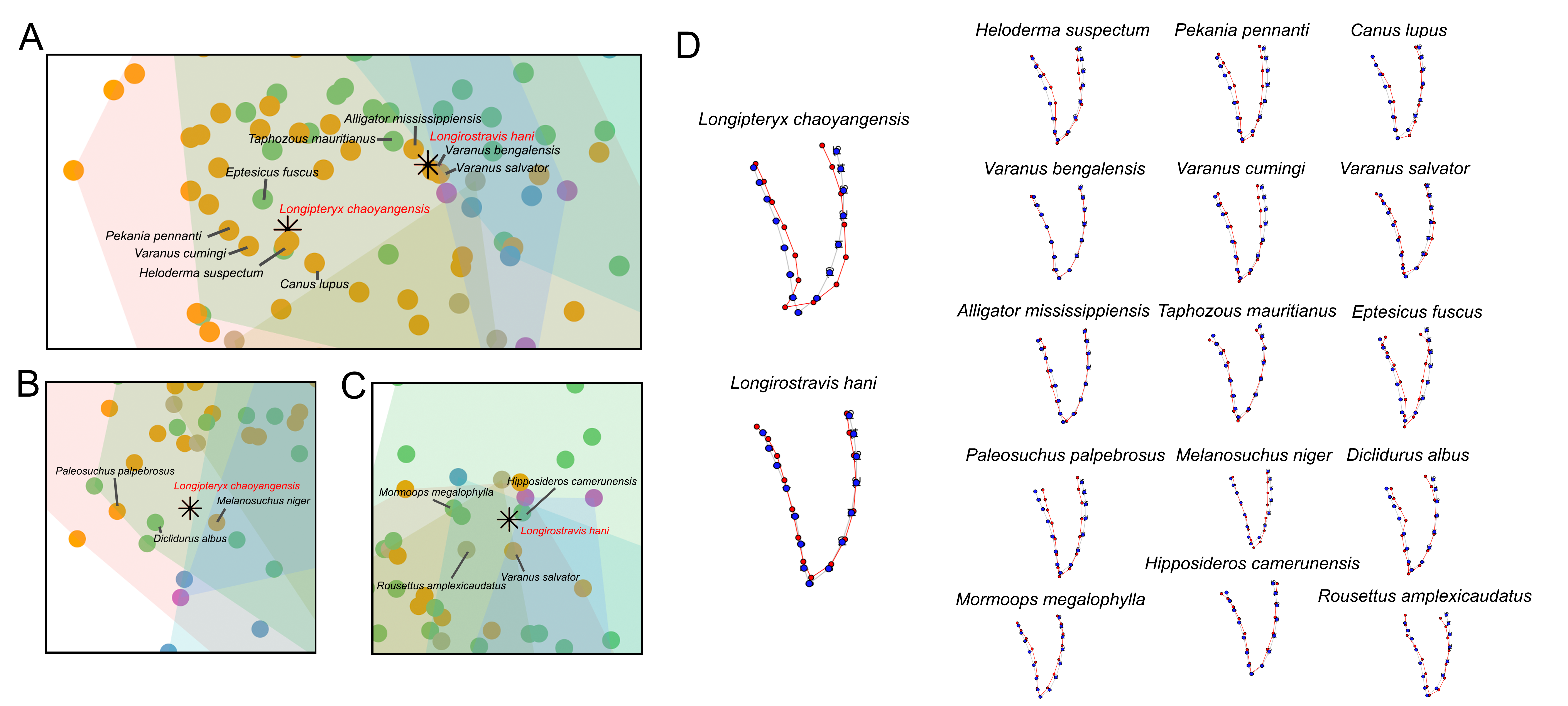

Supplement: Supplemental Information 4 — (A) Magnified section of PC1 × PC2 analysis with closely associated taxa compared to two sampled longipterygids. (B) Magnified section of PC1 × PC3 analysis with closely associated taxa compared to Longipteryx chaoyangensis. (C) Magnified section of PC1 × PC3 analysis with closely associated taxa compared to Longirostravis hani. (D) GM morphologies of sampled longipterygids and labeled taxa from A–C. As in Figure 3, mean tooth morphology is represented by blue and the specific taxon representation in red. All taxon sampled and protocols used can be found in the Supplemental Information. [file peerj-11-15139-s004.png]
